# Species-specific N-glycan patterns in animal and human breast milk samples

**DOI:** 10.3389/fnut.2025.1597284

**Published:** 2025-09-30

**Authors:** Davide Ret, Salvatore Alessio Gentile, Johanna Rohrhofer, Larissa Koidl, Francesca Cozzi, Eva Untersmayr

**Affiliations:** ^1^Research Unit Macromolecular Chemistry, Institute of Applied Synthetic Chemistry, TU Wien, Vienna, Austria; ^2^Institute of Pathophysiology and Allergy Research, Center for Pathophysiology, Infectiology and Immunology, Medical University of Vienna, Vienna, Austria

**Keywords:** milk glycosylation, N-glycans, sialic acid, N-glycolylneuraminic acid (Neu5Gc), N-acetylneuraminic acid (Neu5Ac)

## Abstract

The main components of milk are protein, fat and carbohydrates. Glycans are complex carbohydrate structures covalently bound to proteins and lipids or exist freely in solutions. The function of glycans in nature is to contribute to structure, energy metabolism and encoding relevant biological information. In this study we have analyzed the content of sialic acids. Moreover, we assigned and compared the N-glycan profile of the milk casein and whey fraction from humans, infant formula, cows, horses, goats, and sheep. Conservation of terminal sialic acids on the glycan structures was achieved by stabilization via methylamidation. Human breast milk exhibitsa unique glycan profile, characterized byhighly fucosylated N-glycans and is rich in N-acetylneuraminic acid (Neu5Ac), while N-glycolylneuraminic acid (Neu5Gc), a sialic acid not synthesized in humans, was not detected. In contrast, other mammalians are characterized by a species-specific N-glycan profile with low amounts of fucosylated glycans and different amounts of Neu5Gc. Sheep’s milk has the highest amount of Neu5Gc, followed by goat’s milk, horse’s milk, and cow’s milk. In infant formula, the concentration of total sialic acid is reduced in comparison to human breast milk and contains approximately 7% of the non-human sialic acid Neu5Gc. Regarding the analysis of N-glycan profiles comparing casein and whey fractions, no differences were observed for human breast milk, only marginal differences for cow, horse, goat and sheep and pronounced differences in infant formula. Our work confirms that human breast milk has a unique N-glycan signature with a distinct glycan composition when compared to all other mammalian milk samples. No traces of the non-human sialic acid Neu5Gc were detected, whereas it was found in varying amounts throughout the other milk samples.

## Introduction

Milk and milk products are important components of the human diet providing access to essential nutrients, such as proteins, fats, carbohydrates, vitamins, hormones and minerals (1). Human breast milk is usually the main nutritional source in the first months of life. After weaning, other milk sources become of more importance.

Eighty-one percentage of the global milk production is sourced from cows, followed by buffaloes (15%), goats (2%), sheep (1%) and camels (0.5%). The remaining share comes from other dairy producing species such as equines and yaks (2). The utilization and importance of milk from different species varies significantly across geographical regions. In most developed countries, the major amount of milk is produced by dairy cows, while about one third of the milk is produced by other species. In geographical regions with harsh environmental and unfavorable climatic conditions, small ruminants are often preferred due to their rapid generational turnover and short pregnancies. This results in an earlier milk production compared to larger animals and provides milk in quantities that are suitable for immediate household consumption (2).

In human breast milk, carbohydrates are highly abundant and are found as free human breast milk oligosaccharides (HMOs) or conjugated to proteins and lipids. Apart from nutritional values, these carbohydrates serve as building blocks for newly synthetized tissues and organs (3). Carbohydrates also play an important role in determining the function and longevity of proteins and lipids. Many naturally occurring sugars (such as galactose, mannose, N-acetyl glucosamine, fucose, N-glycolylneuraminic acid (Neu5Gc), N-Acetylneuraminic acid (Neu5Ac) and many others) are combined to create a variety of unique glycan structures. These oligosaccharides, but also phosphorylated glycans or glycosaminoglycans, can be bound to proteins covalently via N-glycosidic bond (N-glycans) or O-glycosidic bond (O-glycans) during post-translational modification. Depending on the species-specific enzyme apparatus and available sugar molecules, the resulting protein glycosylation is unique to each species. Therefore, the glycosylation patterns of secreted milk proteins are also expected to be species specific. The glycans attached to milk proteins are responsible for various functions. In newborns, glycans play a nutritional and protective role. In humans, rapid brain growth requires large amount of building blocks such as Neu5Ac for neuronal growth. Failure to meet nutritional needs during this crucial period of brain growth has significant consequences for cognitive development (3). Surface glycans also act as receptors for pathogens in addition to showing anti-inflammatory effects by limiting the antigenicity and immunogenicity of the proteins they are bound to (4–6). Sialic acids play an important role in inflammatory processes of the body. Humans are characterized exclusively by Neu5Ac. However, an exchange on the cell surface of Neu5Ac is possible after Neu5Gc ingestion with diet (7, 8). Surface bound Neu5Gc can lead to recognition of the structure as non-self, subsequently initiating an immune response against this “xeno-autoantigen” (9). Therefore, accumulation of Neu5Gc might result in chronic inflammation (10). Moreover, a Neu5Gc-rich glycocalyx was described to be associated with cancer formation (7). For this reason, understanding the detailed glycan composition and structure of dairy products is crucial, given their fundamental role in global nutrition.

Several attempts have been made to characterize the milk glycome (11, 12). However, due to various methodological limitations, the terminal sialic acids of N-glycans have not been preserved during investigations, resulting in an incomplete analysis. Furthermore, evaluation of the whey fraction is of fundamental importance, given its widespread use in the food processing industry (13, 14).

As glycans introduced by diet have been suggested to influence human health due to potential alterations of human glycosylation pattern, we aimed to determine the N-glycan profile of the casein and whey fraction and to quantify sialic acid levels of milk from different sources. Particular emphasis was placed on stabilizing the labile sialic acid residues on the antennary position of the N-glycans by methylamidation (15) allowing the complete conservation of the glycan structure. However, due to the method applied and the focus of this study on sialic acid composition, we did not analyze the conformation of the linkage (isomers).

## Materials and methods

N-acetylneuraminic acid (Neu5Ac) was obtained from Carbosynth (Berkshire, UK), N-Acetylglucosamine (GlcNac) was provided by Alfa Aesar (Kandel, Germany), 4,5-dimethylbenzene-1,2-diamine (DMBA) and methylamine hydrochloride were provided by Sigma-Aldrich (Darmstadt, Germany), 4-(4,6-Dimethoxy-1,3,5-triazin-2-yl)-4-methyl-morpholiniumchlorid (DMTMM) was prepared as previously described (16), maltodextrine (Agrana, Austria), cow’s milk (Leichte Murauer Bergbauern, Milfina Haltbare Vollmilch, Milfina Leichte Haltbarmilch), sheep’s milk (Schafmilch Zurück zum Ursprung Bio Hofer), goat’s milk (Ziegenmilch Zurück zum Ursprung Bio Hofer) were bought in a local supermarket (Vienna, Austria). Cow colostrum was obtained from Allcura (Germany). Organic raw milk was directly purchased from a local milk producing farm in Lower Austria. Horse’s milk powder was bought from Green Line Nutritheke (Röttenbach, Germany). Infant formula bought from Aptamil HA Pre (Frankfurt, Germany) and Hipp Combiotik HA PRE (Gmünden, Austria). Previously frozen human milk samples were collected from bottle feeding leftovers from 3 healthy breastfeeding adult women. Milk was collected 3 months after birth. All the liquid milk samples were stored at −20 °C until further processing.

### Extraction and quantification of milk whey and casein proteins

Milk samples from animals (cows, goats, horses and sheep) were diluted in 1:2 ratio (0.5 mL of fresh sample plus 1.0 mL of deionized water). Due to the lower fat content, human breast milk samples were fractioned undiluted. All samples were centrifuged at 10,000 g for 30 min at 4 °C, resulting in three different fractions: precipitate (casein), aqueous middle fraction (whey) and top layer (milk lipids). To skim the samples, the lipid layer was removed accurately using a spatula. The aqueous supernatant was transferred into a new tube. Both protein fractions were centrifuged again in separate tubes at 10,000 g for 10 min at 4 °C. Contaminating fractions were removed, resulting in pure casein and whey fractions. To delipidate and precipitate the protein fractions, 600 μL 2-propanol and 900 μL hexane were added to each sample, cooled down on ice and gently homogenized for 60 min to improve protein precipitation. Then, the samples were centrifuged at 10,000 g for 10 min, obtaining three clearly visible fractions consisting in a top layer (organic phase containing fat residues), the middle layer (aqueous/alcoholic phase) and the precipitated glycoproteins in the bottom layer. After removing the supernatants, the protein pellet was dried at 45 °C in a vacuum dry oven (Binder FDL 115, Germany) to evaporate the solvent residues until constant weight. The weight of the casein and whey was determined.

### Quantification of total Neu5Ac and Neu5Gc via HPLC-FL

**Figure 1:**
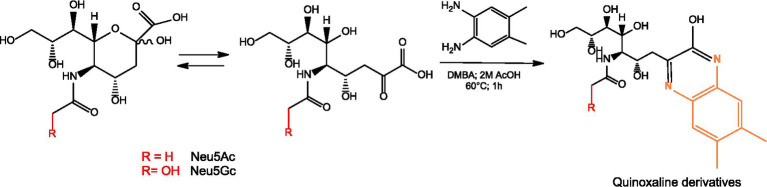
DMBA derivatization of sialic acid and formation of fluorescent quinoxaline derivatives.

Quantification of Neu5Gc and Neu5Ac was performed by HPLC (Agilent 1,100, Germany) with fluorescence (FL) detector (Shimadzu RF-551, Japan). 100 μL of samples, standard and control were introduced into 1.5 mL reaction tubes. O-acetyl groups were hydrolyzed by adding 10 μL 1 M NaOH at RT for 30 min. To cleave the sialic acid from the glycoconjugate, samples were treated with 90 μL of concentrated acetic acid for 2 h at 80 °C. The samples were centrifuged at 10,000 g for 20 min to remove the precipitates. DMBA working solution was prepared by dissolving 32 mg of DMBA in 1.15 mL of concentrated acetic acid and diluted to 10 mL with water. In a 1.5 mL reaction tube, 250 μL of DMBA working solution and 50 μL of the supernatant were mixed. The samples were incubated for 1 h at 60 °C. 150 μL of ammonia at 30% was added. 100 μL of methanol was added to increase the solubility. The solution was transferred to an HPLC vial and measured. The DMBA-derivatized samples were analyzed on an Agilent 1,100 HPLC system using a RP C18 250×4.6 mm column from Applichrom at 25 °C. The fluorescence was detected at 432 nm, using excitation at 379 nm. The injection volume was set to 10 μL (Figure 1).

The analysis was performed using a gradient time program described in Table 1.

**Table 1:** HPLC RP-C18 gradient.

Time (min:sec)	Metanol (%)	Water with 0.1% TFA
1	20	80
10	30	70
13	60	40
13:10	99	1
18	99	1
18:10	20	80
25	20	80

**Figure 2:**
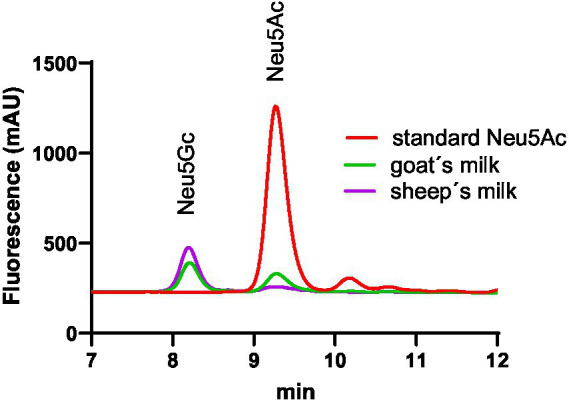
Example of RP chromatograms of DMBA labeled sialic acid. The retention time of Neu5Gc labeled sialic acid was 8.4 min and was 9.4 min for Neu5Ac.

In Figure 2 examples of the chromatogram of the Neu5Ac-DMBA and Neu5Gc-DMBA fluorescent adducts are shown. It was possible to observe a very good separation of the two main analytes.

### N-glycan analysis

#### Modification with methylamine

**Figure 3:**
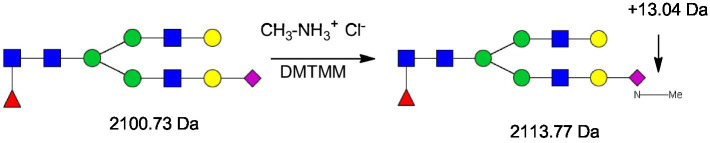
Methylamidation of sialic acid increased the molecular weight of the glycans by 13.04 Da. GlcNAc, N-acetylglucoseamine [blue square]; (mannose) [green circle], galactose [yellow circle]; Neu5Ac, 5-N-acetylneuraminic acid [purple diamond]; and Neu5Gc, 5-N-glycolylneuraminic acid [white diamond].

Four mg of solid glycoprotein (whey or casein) was treated with 200 μL of a solution of DMTMM 0.25 M, methylamine hydrochloride 0.44 M in 10 mM phosphate buffer at pH 6. The reaction tube was vortexed and incubated at 70 °C for 1 h. 200 μL of MeOH was added, vortexed and centrifuged at 10,000 g for 10 min. This centrifugation step was repeated twice to remove excess salts and byproducts of the glycoprotein derivatization (Figure 3).

### N-glycan hydrolysis

The precipitate was suspended with 100 μL of denaturing solution consisting of phosphate buffer solution 20 mM at pH 6.2, 0.1 M dithiothreitol (DTT), 0.2% of SDS and 12 μL NP-40 (10% in water). 0.5 μL of enzyme PNGase F was added to the sample and incubated for 24 h at 37 °C. Thereafter, the sample was centrifuged at 11,000 g for 10 min. 100 μL of the supernatant was transferred into a new reaction tube.

### Purification with HILIC-SPE

N-glycan purification and sample concentration were performed using cotton HILIC-SPE micro extraction (17) as previously described (15). Briefly, to 100 μL of supernatant 488 μL of acetonitrile (ACN) was added to adjust the concentration of ACN to 83% in volume, which was required for HILIC purification. Three mg of cotton were inserted into a 200 μL polypropylene pipette tip and washed three times with 50 μL of water. Subsequently, the tip was equilibrated three times with 50 μL ACN 83%. Aliquots of 100 μL were transferred in a new reaction tube and pipetted up and down 10 times. Afterwards, the tip was washed with 100 μL of ACN 83% three times. Glycans were eluted from the cotton by pipetting five times up and down with 5 μL of water. The samples were stored at −20 °C until further analysis.

### MALDI measurement

A 2,5-dihydroxybenzoic acid (DHB) matrix solution was prepared by dissolving 5 mg of 2,5-DHB to 500 μL of ACN and diluting it in 500 μL of 10 mM NaCl solution. 0.5 μL of matrix solution was mixed with 0.5 μL of purified glycan sample. One μl of the matrix-glycan-mix was spotted on a MALDI-MS steel target and dried at room temperature. For a typical MALDI analysis, solutions of a sample (concentration ∼10 μM) and matrix (concentration ∼10 mM) were pre-mixed and a small volume (∼1 μL) was applied directly to the target (18). The shape of the matrix crystals was uniformed by addition of 0.2 μL EtOH causing a rapid crystallization. MALDI/MS measurements were performed using a MALDI-TOF Ultraflex (Bruker Daltonics, Germany), operating in reflection positive ion modes. The instrumental conditions employed to analyze molecular species in the m/z range 1,000–3,500 were: ion source 1: 20.00 kV; ion source 2: 18.00 kV, lens: 9.00 kV, pulsed ion extraction: 140 ns; laser power: 85%. Each sample was recorded with 2000 shots at 200 Hz. A maltodextrin ladder was used as external mass calibration.

### Data analysis

The obtained spectra were exported as text format using flex analysis version 2.4 (Bruker Daltonics, Germany). The mass spectra were recalibrated using a maltodextrin ladder as external standard with mMass 5.5.0 software.^1^ Glycan structures where assigned using the GlycoWorkbench 2.1 software (19). The database used for the assignment with the methylamidated sialic acid can be found in the Supplementary Table S2. The data were plotted using GraphPad prism 8 software (GraphPad Software, Massachusetts USA). MS spectra were normalized to the glycan 1,663 Da and put to an intensity of 100. All glycan assignments were made following a specific tolerance (<1 Da). For integration the first isotope was used.

## Results

Three cow’s milk samples from different producers were analyzed separately. The results were averaged and presented as a single result. The same procedure was applied to the human breast milk samples.

### Casein and whey protein content in different milk samples

Table 2 summarizes the different amounts of casein and whey proteins present in 1 mL of each milk sample. The highest total protein content was observed in sheep’s milk and horse’s milk, the lowest in human breast milk, infant formula and cow’s milk colostrum. Casein proteins were the main components in cow’s milk, horse’s milk, goat’s milk, sheep’s milk, and infant formula. The main protein component of human breast milk and colostrum was whey proteins (Table 2).

**Table 2:** Mg of whey and casein in 1 mL of milk sample.

Number	Sample	Casein (mg/ml)	Whey (mg/ml)	Total protein (mg/ml)
1	Human breast milk	5	11	16
2	Infant formula*	6	4	9
3	Cow’s milk	95	9	104
4	Horse’s milk*	210	7	217
5	Goat’s milk	103	17	120
6	Sheep’s milk	155	9	165
7	Cow’s milk raw	102	10	112
8	Cow’s milk colostrum*	6	7	14

*Milk powders reconstituted as described by the manufacturer of cow’s milk colostrum 100 mg/mL and horse’s milk 100 mg/mL.

The whey protein fraction was the major component in cow’s colostrum and human breast milk, while casein was the primary protein fraction in all other milk samples. Therefore it was relevant to analyze the glycosylation patterns of whey and casein fractions separately for all studied milk types.

### Total sialic acid quantification

**Figure 4:**
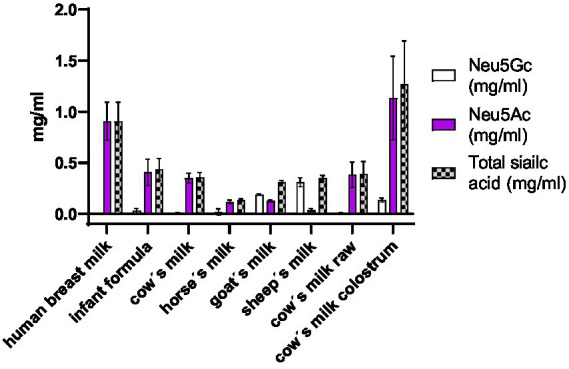
Concentration in mg/ml of Neu5Gc, Neu5Ac and total sialic acid in different milk samples.

The concentration in mg/ml of Neu5Ac and Neu5Gc in milk was measured by HPLC-FL DMBA. With this method, it was possible to accurately quantify the total Neu5Ac and Neu5Gc concentrations (Figure 4). The amount of sialic acid in the milk depends on the lactation period as was also previously reported in the literature (20, 21). Evaluation of cow’s milk colostrum revealed the highest concentration of sialic acid. The human breast milk analyzed in this study was from leftovers collected after a lactation period of 3 months and came from 3 different mothers. Cow’s milk, horse’s milk, goat’s milk, sheep’s milk, came from pooled milk of many animals with different lactation periods (all mature milk). Cow’s milk colostrum and human breast milk showed the highest concentration of total sialic acid with a concentration of around 1 g/L. The main difference observed between these two samples was the presence of the Neu5Gc. Human breast milk contained no detectable traces of Neu5Gc. Cow’s milk colostrum contained 11% of Neu5Gc.

**Figure 5:**
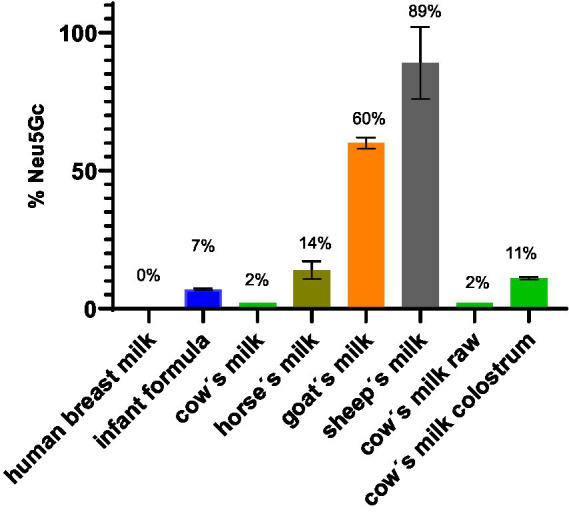
Weight % of Neu5Gc of total sialic acid in milk.

The highest amount of Neu5Gc was observed in sheep’s milk (89%) followed by goat’s milk (60%). Our results obtained for goat’s milk were comparable to values available in the published literature (22). Less Neu5G was detected in horse’s milk (14%), cow’s milk colostrum (11%), infant formula (7%) and cow’s milk (2%). In human breast milk no Neu5Gc was detected (Figure 5). No difference was observed between raw, unprocessed cow’s milk and processed cow’s milk.

### N-glycan analysis of milk samples

**Figure 6:**
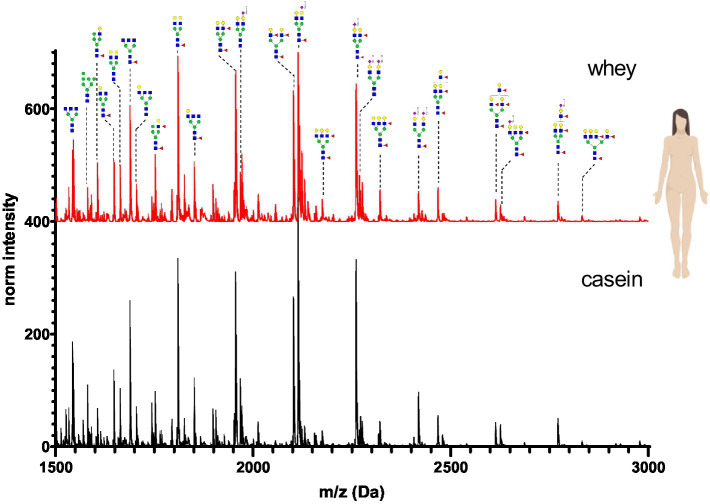
MALDI-TOF/MS spectra of the methylamidated N-glycan profile of human breast milk casein and whey fraction. The spectra intensity was normalized to the signal at 1663 Da.

**Figure 7:**
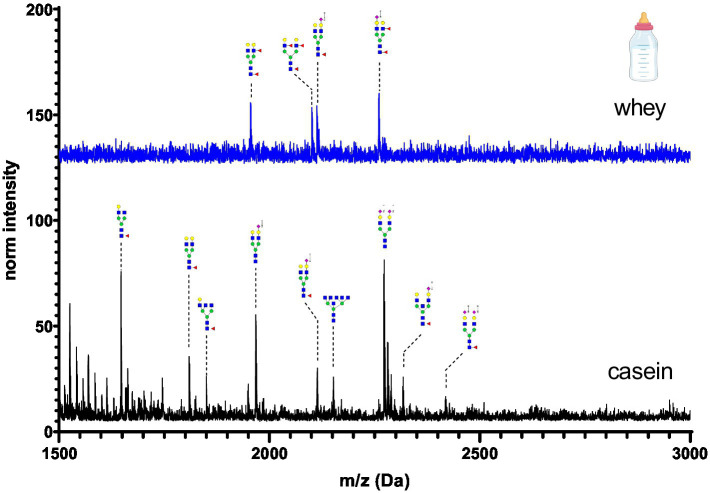
MALDI-TOF/MS spectra of the methylamidated N-glycan profile of infant formula casein and whey fraction. The spectra intensity was multiplied by 100 and not normalized due to the absence of the normalization signal at 1663 Da.

**Figure 8:**
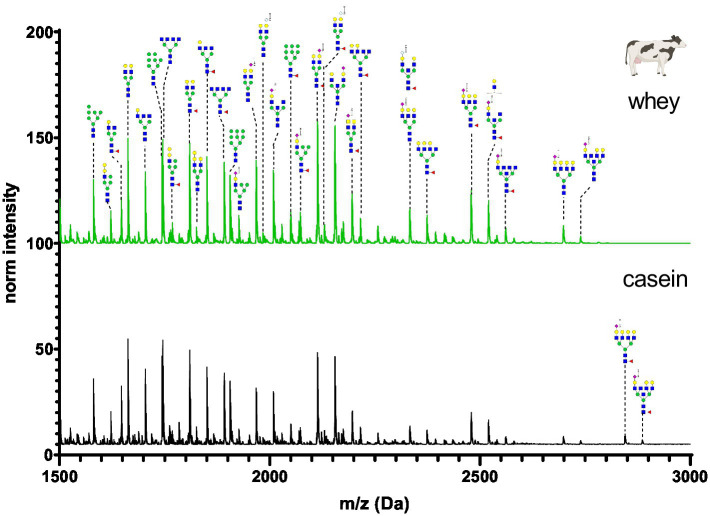
MALDI-TOF/MS spectra of the methylamidated N-glycan profile of cow’s casein and whey fraction. The spectra intensity was normalized to the signal at 1663 Da.

**Figure 9:**
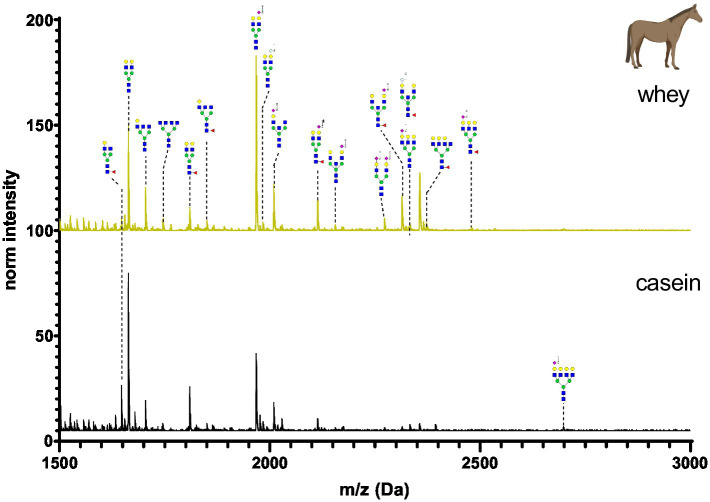
MALDI-TOF/MS spectra of the methylamidated N-glycan profile of horse’s casein and whey fraction. The spectra intensity was normalized to the signal at 1663 Da.

**Figure 10:**
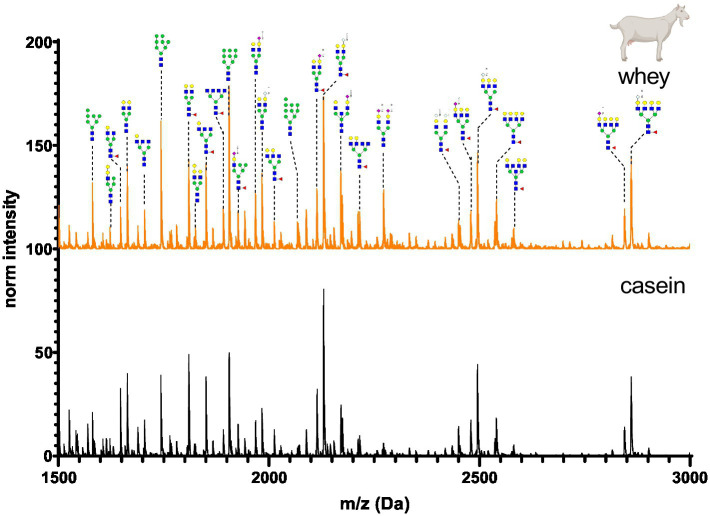
MALDI-TOF/MS spectra of the methylamidated N-glycan profile of goat’s casein and whey fraction. The spectra intensity was normalized to the signal at 1663 Da.

**Figure 11:**
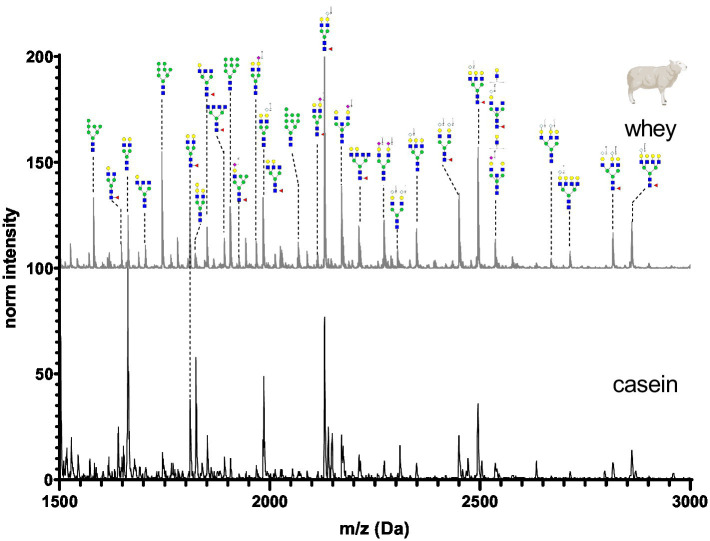
MALDI-TOF/MS spectra of the methylamidated N-glycan profile of sheep’s casein and whey fraction. The spectra intensity was normalized to the signal at 1663 Da.

The N-glycan mass spectrum annotation was performed using the software GlycoWorkbench 2. A new library with more than 140 substances was created. In the library, N-glycans were underivatized at the reducing end (free reducing end), bear one sodium ion (+22.99 Da) and all sialic acid were methylamidated (+13.04 Da, see Figure 2). Whey and casein milk fractions were analyzed separately for each milk sample. Figures 6–11 show a comparison of the milk N-glycan profile of casein and whey from different species. The most common glycans are depicted as a structure.

N-glycans in human breast milk were characterized by a high proportion of complex bi- and tri-antennary N-glycans. Mono- and di-sialated N-glycans (Neu5Ac-terminal) were present. No traces of the non-human sialic acid Neu5Gc were observed. Human breast milk was characterized by high levels of core fucosylated and antennary fucosylated glycans. The N-glycan composition was very similar in casein and whey. Only minor differences in the intensity of detected glycans were observed between the two fractions (Figure 6).

Infant formula casein and whey fraction N-glycans are shown in Figure 7. A cow’s milk based infant formula was chosen for this investigation as this is the most common substitute for human breast milk. In comparison to all other samples, the intensity of glycans was two-fold reduced. This can be explained by the processing of the milk powder. Milk-based formulas are often hydrolyzed, which may result in a loss of glycans or glycopeptides from the protein backbone. The whey fraction was characterized by presence of only four glycans. The casein fraction was characterized by bi-antennary glycans with one or two sialic acids.

The N-glycan profile of cow’s milk casein and whey fractions (Figure 8) was characterized by a high proportion of asialo N-glycans and a lower amount of fucosylated glycans. Only monosialated N-glycans were observed and the majority contained Neu5Ac. Neu5Gc was present only in small amounts. Various alpha-galactose structures were found in cow’s milk. Alpha-galactose structures are known as triggers for allergic reactions. To assess if the heat treatment (pasteurization) could affect the N-glycans, organic raw farm milk was analyzed before and after pasteurization. The N-glycan profiles of the milk casein and whey are largely similar, with differences primarily observed in high-molecular-weight glycans. Comparing the N-glycan profile of organic raw cow’s milk coming from a small farm with few animals to the pooled commercial cow’s milk, a difference in the glycan intensity profile was observed (Supplementary Figure S2).

Horse’s milk glycan profiles of casein and whey are depicted in Figure 9. The most abundant glycans had one Neu5Ac at the antennary position. Very low amounts of fucosylated N-glycans were observed. The used horse’s milk samples originated from a reconstituted dry milk powder. The processing might have led to the loss of some glycans. N-glycan composition was very similar in casein and whey horse’s milk sample, but differences were observed for high molecular weight glycans.

Goat’s milk casein and whey N-glycan profiles are shown in Figure 10. N-glycans were characterized by similar amounts of Neu5Gc and Neu5Ac in the antennary position. The whey and casein profiles were almost identical, but marginal differences were observed in intensity of low molecular weight glycans.

Sheep’s casein and whey was characterized by a high amount of bi-, tri- and tetra-antennary N-glycans. Most of the glycan structures contained one or two Neu5Gc at the antennary position. Neu5Ac was present in very low amounts in sheep’s milk. Minor differences were observed in the intensities of low molecular weight mannosylated glycans.

**Figure 12:**
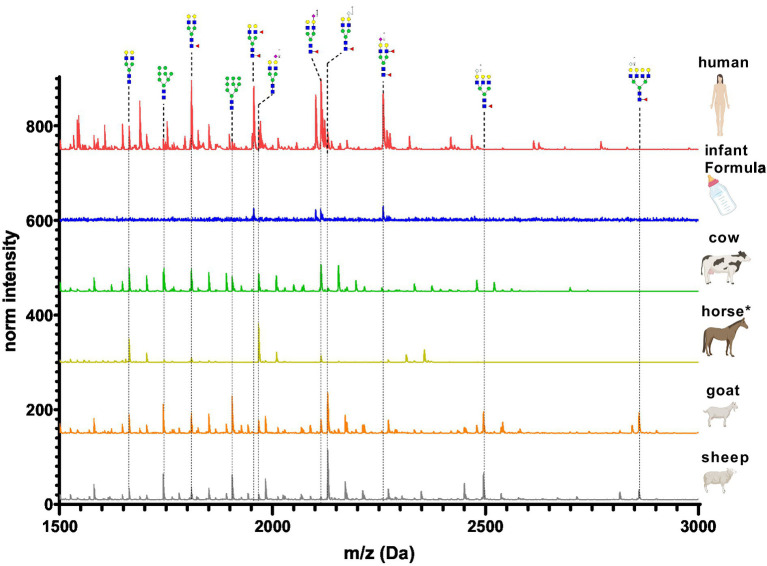
Differences in whey milk MALDI-TOF/MS spectra of methyl amidated N-glycan profiles from human breast milk, infant formula, goat’s, horse’s, cow’s and sheep’s milk. The glycans with the highest difference between the samples are indicated and labeled.

**Figure 13:**
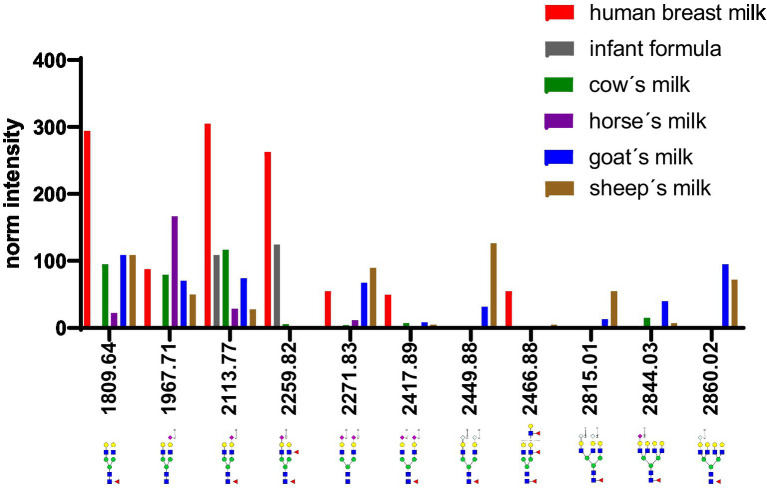
Comparison of intensity of selected N-glycans from the whey fraction of different milk samples. All glycans were normalized to the glycan with a molecular weight of 1,663 Da. See Supplementary Table S3 for more information.

Figure 12 depicts the whey N-glycan profile of human breast milk, infant formula, cow’s, horse’s, goat’s and sheep’s milk showing the heterogeneity of the different N-glycans in milk from different milk sources. In this study, we are able to directly compare the glycan profiles of different milk species using a uniform analytical method. This contrasts with previous studies, in which only one or a limited number of species were analyzed under comparable conditions (Figure 13) (23, 24).

## Discussion

### Method improvement

The procedure used in this study enabled the measurement of all N-glycans including that with sialic acids in the antennary position. This allows for obtaining the complete N-glycan profile of the different milk samples. Through stabilization and charge neutralization of sialic acid, our methodology enabled a complete conservation of the glycan structures and detection in MALDI positive modes. This contrasts with other methods in which sialic acids are often lost due to their instability at low pH during sample preparation or the use of inappropriate polarity in MALDI mass spectrometry measurement (12). Another advantage of our method is the solvent used for the modification reaction. Using water as solvent for the methylamidation reaction ensured a homogeneous derivatization of the sample.

### Whey and casein factions

To the best of our knowledge, our study is the first to measure milk N-glycans in whey and casein separately. Casein is the main fraction of milk used for human nutrition and cheese processing (Table 2). In contrast, whey is the main fraction of the human breast milk and cow colostrum. Therefore, it was important to analyze the glycosylation of whey and casein milk fractions separately.

### Species specific sialic acids

Each milk has a characteristic total amount of sialic acids and ratio of Neu5Ac and Neu5Gc. Between the different milk samples the milk with the highest total sialic acid content is human breast milk (0.91 g/L) followed by cow’s milk (0.38 g/L), sheep’s milk (0.35 g/L) goat’s milk (0.31 g/L) and horse’s milk (0.14 g/L). The weight percentage of the non-human sialic acid Neu5Gc found in human breast milk is 0%, in cow’s milk 2%, in sheep’s milk 89%, in goat’s milk 60%, in horse’s milk 14% (Figure 5). Based on the amount and kind of sialic acid, cow’s milk is most similar to the human breast milk composition. Sheep’s milk exhibited the lowest similarity to human breast milk in terms of sialic acid composition. There is no significant difference regarding the sialic acid amount and composition between raw and pasteurized cow’s milk. This suggests that pasteurization treatment does not affect sialic acid composition.

### Species specific N-glycans

For each species, the N-glycosylation of the casein protein fraction and the corresponding whey protein fraction appeared almost identical. Human breast milk N-glycan profiles revealed a much higher number of glycan structures in comparison to milk from other species. The higher number of peaks was observed near the N-glycans bearing Neu5Ac. This might be due to the natural occurring post-glycosylation modifications such the O-acetylation. In comparison to all other milk samples, human breast milk N-glycans showed heavily fucosylated glycans. With regards to glycan composition, all non-human milk samples had similar sugar composition (N-acetylgucosamine, mannose, galactose, fucose, N-acetyl neuraminic acid and N-glycolyl neuraminic acid). Human breast milk was the only exception bearing no Neu5Gc. However, in our study the exact glycan structure conformation of the glycosidic bond and exact position of the sugars in the glycan was not investigated. The glycan composition of human and cow’s milk found in this study was similar to the glycan composition found in the work of Nwosu et al. (25) using the complementary analytical method of LC–MS. N-glycan profiles of casein and whey show very similar compositions only in human breast milk samples. The most pronounced difference was observed in infant formula, where only four glycans were detected in the whey fraction. The casein infant formula fraction showed diverse N-glycans characterized by bi-antennary glycans with one or two sialic acids. For all other milk samples only marginal N-glycan differences were observed especially in the higher molecular weight.

### Milk in infant diet

**Figure 14:**
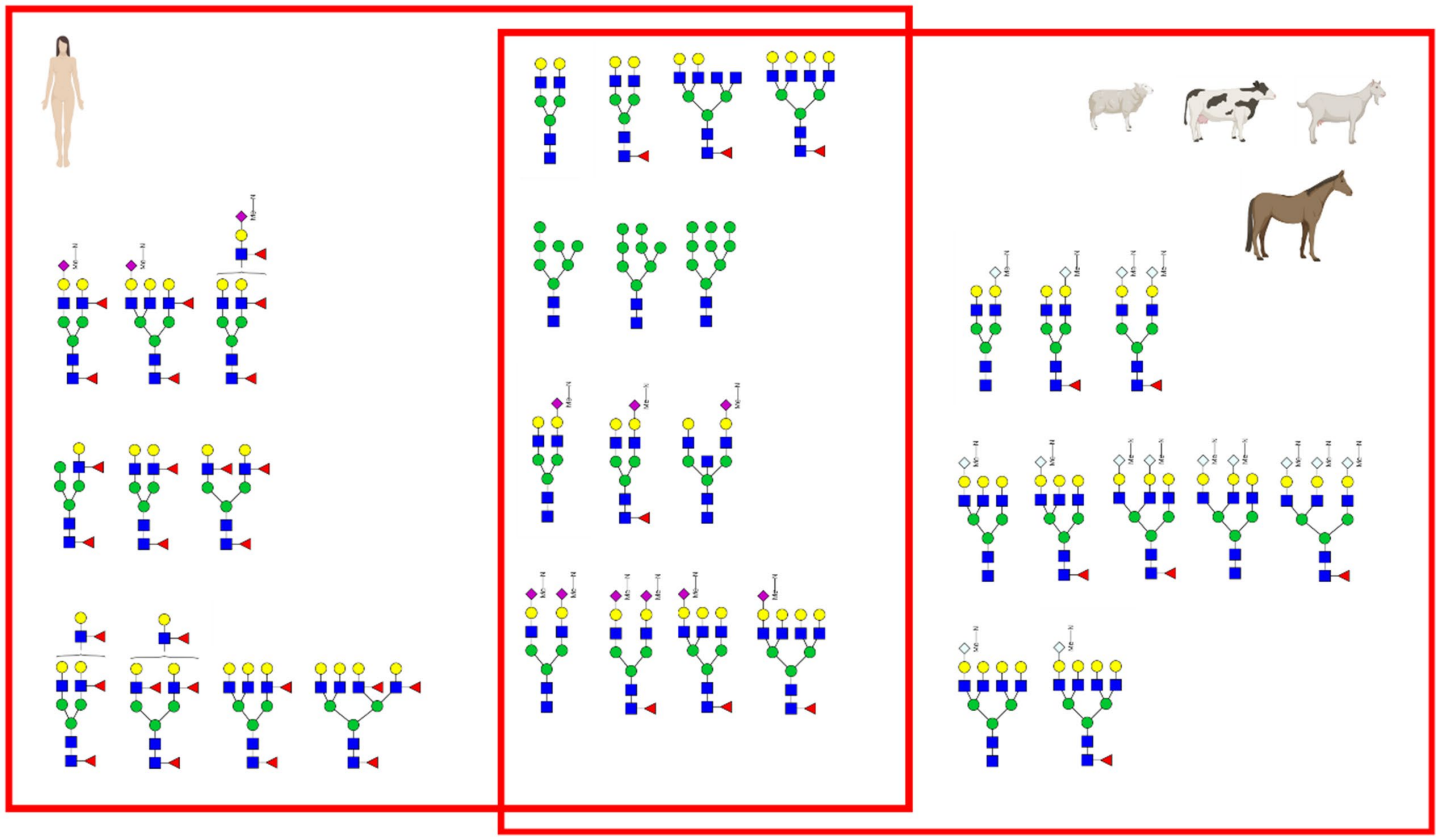
Venn plot of principal glycans in the milk. Only a few glycans were found to be common between the different species.

The most prominent difference between human breast milk and all the other animal derived milk samples is the complete absence of the non-human sialic acid Neu5Gc (Figure 5). Additionally, the concentration of Neu5Ac in human breast milk is very high (0.9 g/L) compared to other milk and milk formula. Human breast milk distinguishes itself from the other mammalian milk by the high concentration of fucose and presence of structurally different fucosylation on the N-glycan antennae (Venn plot Figure 14). Milk fucosylation plays a big role in the gastrointestinal tract of infants, especially in terms of adhesion of pathogens. Fucosylated milk glycans bind bacterial or viral adhesin preventing the adhesion of the pathogens to gastrointestinal epithelial cells (26).

Milk-based infant formula composition depends mostly on the animal source of the milk. The analysed infant formula was a cow’s milk based infant formula. The total sialic acid amount was 0.4 g/L, with the non-human sialic acid being present in 7%. Due to the different protein composition of milk from other sources such as goat’s, sheep’s and horse’s is available commercially as alternatives with a reduced milk protein cross-reactivity (27). In these milk sources, higher concentrations of the non-human sialic acid were found. Due to the chemical similarity of the human Neu5Ac and the non-human Neu5Gc, the latter may be integrated in human glycans and accumulate in the body, which may elicit an inflammatory immune response (7) even early in life.

### Use of dairy products in human nutrition

The importance and presence of dairy animals varies significantly depending on the geographical region. In countries with scarce grazing and unfavorable climate conditions (2) most of the dairy products come from sheep and goats, while in most industrial countries the main dairy source is cow’s milk. In this study the milk of the most common Austrian dairy animals, as well as human breast milk and infant formula was analysed. With regards to immune-nutrition within the human diet the most interesting effect of glycans in milk are: (i) presence of non-human sialic acid and (ii) structural and conformational effects of glycans (e.g., of alpha galactose). After ingestion, milk is subjected to acidic pH and digestive enzymes. Moreover, microbial neuraminidase cleaves sialic acids from glycans. At acidic pH, sialic acid is hydrolysed from the glycans, as we have observed in our previous study (15). Due to its small dimension, the free glycolyl neuraminic acid is unable to produce inflammatory effects, although it does not belong to the human body during this step. Due to the integration of Neu5Gc on the human glycocalyx, a chronic low-grade inflammation process may be initiated (7, 8). Due to the effects of the interplay between glycolyl neuraminic acid and human immune stimulation, possible implications must be considered in immune-nutrition.

There are several limitations to the current study. Due to the focus on sialic acid stabilization for detection, it was not possible to also analyse the conformational isomers. Moreover, we had a limited number of commercial sources for goat’s, sheep’s and horse’s milk, which might result in a bias in the analysis. Moreover, it was beyond the scope of this manuscript to evaluate the reasons for differences in the N-glycan profiles among different species, which is a highly relevant topic for future research.

## Conclusion

In our study we were able to perform a comprehensive analysis of sialic acid and N-glycans across various milk types. Our approach preserved glycan structures, particularly under MALDI positive modes, where traditional methods often fail due to the instability of sialic acids at low pH. The method used not only ensured more accurate profiling but also emphasized the importance of solvent choice in order to achieve homogeneous derivatization. Our analysis revealed distinct glycosylation patterns in milk from different species, with human breast milk exhibiting unique characteristics, such as the absence of Neu5Gc and a higher concentration of fucosylated glycans. These findings have significant implications for infant nutrition, particularly in the formulation of milk-based products, where the presence of non-human sialic acids like Neu5Gc could have potential long-term health impacts.

## Data Availability

The original contributions presented in the study are included in the article/Supplementary material, further inquiries can be directed to the corresponding author.

## Footnotes

^1^ www.mmass.org
