# Supplementary material for: Species-specific N-glycan patterns in animal and human breast milk samples
Source: Front Nutr. 2025 Sep 30;12:1597284. doi: 10.3389/fnut.2025.1597284 (PMC12518109; doi:10.3389/fnut.2025.1597284)
Supplement: Supplementary file 1 [file supplementary_material.docx]

Supplementary Material

**Supplementary Table 1:** Sialic acid concentrations of different milk samples

| Sample | Neu5Gc (mg/ml) | SD | Neu5Ac (mg/ml) | SD | Total sialic acid (mg/ml) | SD | % Neu5Gc of total sialic acid |
| --- | --- | --- | --- | --- | --- | --- | --- |
| human breast milk | 0.000 | 0.000 | 0.907 | 0.186 | 0.907 | 0.186 | 0 |
| infant formula* | 0.031 | 0.021 | 0.406 | 0.130 | 0.436 | 0.107 | 7 |
| cow milk | 0.009 | 0.002 | 0.349 | 0.048 | 0.358 | 0.046 | 2 |
| horse milk * | 0.020 | 0.031 | 0.116 | 0.019 | 0.136 | 0.012 | 14 |
| goat milk | 0.187 | 0.006 | 0.127 | 0.009 | 0.314 | 0.014 | 60 |
| sheep milk | 0.309 | 0.044 | 0.038 | 0.016 | 0.347 | 0.029 | 89 |
| cow milk raw | 0.008 | 0.002 | 0.384 | 0.123 | 0.391 | 0.121 | 2 |
| cow milk colostrum* | 0.135 | 0.019 | 1.134 | 0.409 | 1.268 | 0.426 | 11 |

*milk powders reconstituted as described by the manufacturer of cow milk colostrum 100 mg/ml and horse milk 100 mg/ml

**Supplementary Table 2.** Internal calibration. All sialic acids carry a methylamidation group to neutralize the charge.

| Glycan Structure | structure | m/z |
| --- | --- | --- |
| Hex4HexNAc2dHex1 | 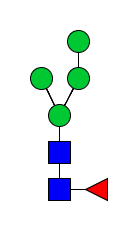 | 1241.43 |
| Hex4HexNAc4dHex1 | 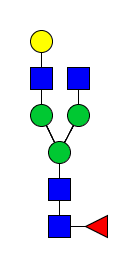 | 1647.59 |
| Hex5HexNAc4NeuAc1 | 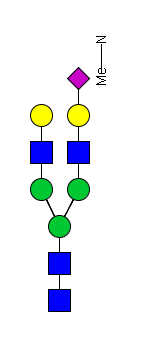 | 1967.71 |
| Hex5HexNAc4NeuAc1dHex1 | 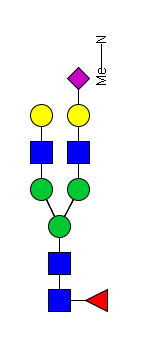 | 2113.76 |
| Hex5HexNAc4NeuAc1dHex2 | 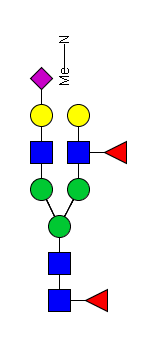 | 2259.82 |
| Hex5HexNAc4NeuAc2 | 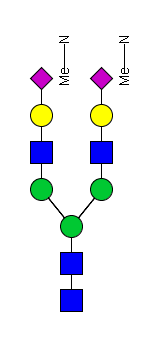 | 2417.89 |
| Hex6HexNAc5NeuAc1dHex3 | 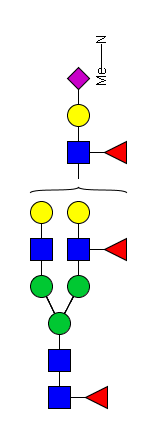 | 2771.01 |
| Hex7HexNAc6NeuAc1dHex3 | 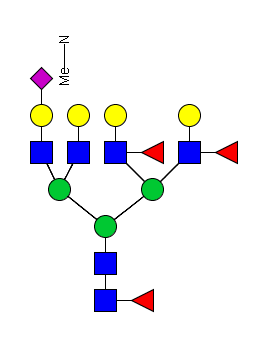 | 3136.14 |

Supplementary Figure 1 MALDI-TOF/MS spectra of the methylamidated N-glycan profile of different the mass spectra of different human milk. The N-glycan in human milk A and C show similar profile

**Supplementary Figure 2** Shows the mass spectra of different commercial cow's milk and organic raw unpasteurized cow's milk. The same glycans can be observed in both cow's milk spectra. The peak intensity of the glycans differs between the different sources.

**Supplementary Table 3.** selected N-Glycans description

| Glycan Structure | structure | m/z |
| --- | --- | --- |
| Hex5HexNAc4  (reference normalization glycan) | 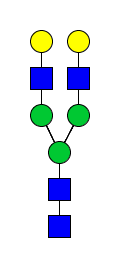 | 1663.58 |
| Hex5HexNAc4dHex1 | 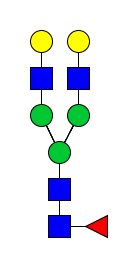 | 1809.64 |
| Hex5HexNAc4NeuAc1 | 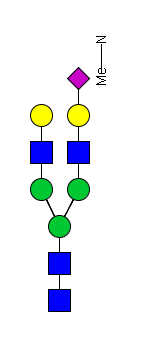 | 1967.71 |
| Hex5HexNAc4NeuAc1dHex1 | 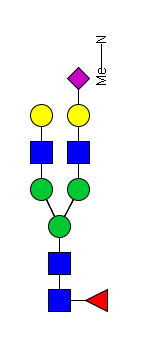 | 2113.76 |
| Hex5HexNAc4NeuAc1dHex2 | 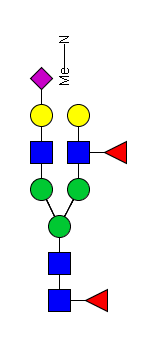 | 2259.82 |
| Hex5HexNAc4NeuAc2 | 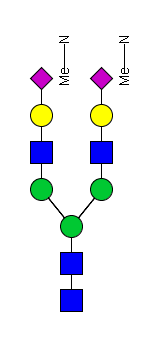 | 2271.83 |
| Hex5HexNAc4NeuAc2 dHex1 | 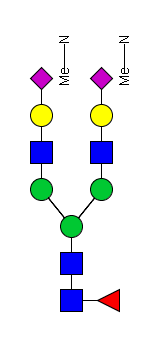 | 2417.89 |
| Hex5HexNAc4NeuGc2dHex1 | 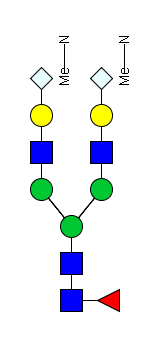 | 2449.88 |
| Hex6HexNAc5dHex3 | 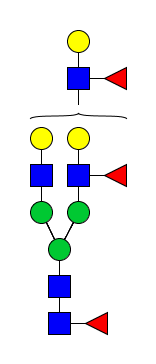 | 2466.88 |
| Hex6HexNAc5NeuGc2dHex1 | 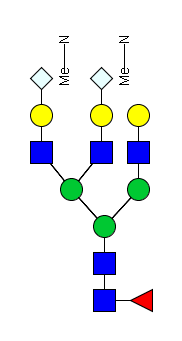 | 2815.01 |
| Hex7HexNAc6NeuAc1dHex1 | 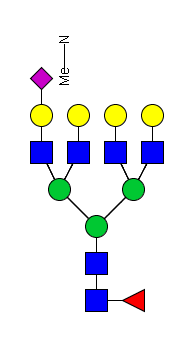 | 2844.03 |
| Hex7HexNAc6NeuGc1dHex1 | 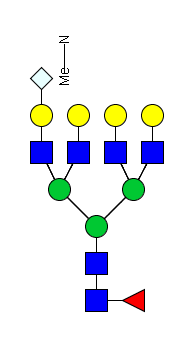 | 2860.02 |
